# Muscle Architecture, Morphology, and Mechanical and Functional Properties of Biceps Femoris Long Head in Professional Soccer Players with a Prior Healed Injured Hamstring

**DOI:** 10.3390/jcm11237222

**Published:** 2022-12-05

**Authors:** Francisco Javier Nuñez, Ramona Ritzmann, Fernando Hernandez-Abad, Juan Carlos Martinez, Luis Suarez-Arrones

**Affiliations:** 1 Department of Sport Sciences, Universidad Pablo de Olavide, 41013 Sevilla, Spain; 2 Department of Sports and Sport Science, University of Freiburg, 79106 Freiburg, Germany; 3 Department of Sport Sciences, European University of the Canary Islands, 38300 Tenerife, Spain; 4 Medical Department, Tottenham Hotspur FC, London N15 4RU, UK; 5 Performance Departament, FC Lugano, 6900 Lugano, Switzerland

**Keywords:** hamstring injury, fascicle length, pennation angle, muscle thickness, Nordic hamstring exercise, isokinetic, football

## Abstract

Objective: The aim of the present study was to compare the fascicle length, pennation angle, muscle thickness and stiffness of the biceps femoris long head, and eccentric hamstring strength between injured dominant limbs, injured non-dominant limbs, uninjured dominant limbs and uninjured non-dominant legs in previously injured players, and between dominant and non-dominant legs in uninjured elite soccer players. Materials and Methods: Twenty elite soccer players participated in this study. Ultrasound imaging and MyotonPRO were used to determine the morphological and mechanical properties of the biceps femoris long head. Isokinetic and Nordic hamstring exercises were used to assess eccentric hamstring strength. Results: Previously injured players showed substantially lower fascicle length and muscle thickness, and significantly higher biceps femoris long head stiffness than uninjured players, without differences between limbs. Conclusion: The morphological and mechanical properties of elite soccer players with hamstring injury history were different from those in uninjured players. The lack of differences between limbs showed that these values are characteristics of individual players that must be considered in the design of programs to prevent BFlh injury.

## 1. Introduction

Hamstring strain injury (HSI) is the noncontact injury in soccer with the highest burden and highest recurrence of a previous identical injury within two months of return to play [1,2]. It is known that HIS in soccer players has a multifactorial etiology [3], and a recent meta-analysis detected up to 21 potential risk factors. The strongest were an advanced age and a history of HSI [4]. The architecture and structure of the musculature encompass risk factors including biceps femoris fascicle length (Fl) [5], muscle–tendon stiffness of the hamstrings [6], and eccentric hamstring strength [7]. Athletes with a history of HSI are 2.7 times more likely to experience recurrences than those without previous injuries, with an elevated risk if the previous and actual HSIs occurred within one season (≈5 times) [4]. However, it is not clear whether the higher prevalence of subsequent injury is due to the architecture (i.e., shorter fascicles), muscle–tendon unit stiffness (i.e., strength–deformation relation), or a low level of eccentric hamstring strength, as a result of declined ability to neuronally innervate the previously injured muscle, or as a consequence of a complex interaction between several risk factors [8,9,10]. Therefore, analysis of the architecture, structure, mechanical properties, and eccentric hamstring strength in the injured and uninjured legs in previously injured players, and in players without previous HSI, is needed to identify the underlying mechanisms and key risk factors.

A meta-analysis published recently on the prevalence of HIS in professional soccer players revealed that fast movements with high eccentric strain in the hamstrings, as occurs in sprint or stretch patterns, are the primary causes of non-contact injuries [11]. Stretch-related injury patterns are caused by hip flexion and simultaneous knee extension, while sprint-related injury patterns occur most often during the late swing phase at high speeds [12]. The biceps femoris long head (BFlh) accounts for 70% of the injuries [13]. Previously injured BFlh muscle exhibits shorter BFlh Fl [14], a decreased eccentric hamstring strength [15] and an increased hamstring muscle–tendon stiffness [6]. It has been hypothesized that longer muscle fibres may diminish injury incidences through reduced stress per sarcomere with reference to a defined muscle–tendon unit strain [14,16]. Additionally, higher eccentric strength may have a protective impact, preventing hamstring muscle strains [5,17]. A previous study has suggested that athletes with short BFlh fascicles of <105 mm have a four times higher risk of suffering an HSI than athletes with longer fascicles of 110 mm [5]. Furthermore, BFlh muscles with a prior strain have shorter fascicles compared to the contralateral leg without a history of HSI [18]. Greater mechanical resistance (i.e., stiffness) to an external force that stretches the muscle was found in elite soccer players with a prior HSI compared to uninjured players, but there were no differences between the injured vs. uninjured legs [6]. Although athletes with previous HSI performed prone leg curls for a shorter duration than uninjured players, and showed an isometric knee flexion force deficit at 15° after return to play [4], the isokinetic strength and Nordic hamstring strength (NHEs) showed no differences among injured and uninjured legs in soccer players [7]. However, Van Dyk et al. [7] combined the results for uninjured limbs of uninjured players and non-injured limbs of injured players, so it is difficult to differentiate between injured and uninjured players in their study.

The aim of the present study was to identify whether elite soccer players with a history of HIS exhibit unilateral differences in Fl, pennation angle, muscle thickness and stiffness, or unilateral deficits in knee flexor hamstring strength (NHEs and isokinetic strength). We hypothesized that previously injured players would have a shorter Fl and higher stiffness in BFlh than uninjured elite soccer players, but there would be no differences between limbs in either group.

## 2. Material and Methods

We executed a cross-sectional cohort study comparing healthy athletes with athletes who had experienced a unilateral HIS that had fully healed. The investigation was performed according to the Declaration of Helsinki. The protocol (ethics and research) was approved by the Committee of the Virgen Macarena and Virgen del Rocío University Hospitals (0398-N-17). Data were anonymized for the analyses to ensure players’ confidentiality.

### 2.1. Participants

Two teams of thirty-five players each from second top European clubs (Super League in Switzerland (taking part in UEFA Europa League) and Serie A in Italy) were analysed. Inclusion criteria for the HSI group were (a) an age >17 years, (b) former HSI of category I-III medically documented by MRI and/or ultrasound, (c) HSI that was fully healed and players medically fully cleared for training and competition for <4 weeks, and (d) no acute orthopaedic, muscular or neuronal injuries/defects. Inclusion criteria for the control group were (a) an age >17 years, (b) bilaterally no HSI, and (c) no acute orthopaedic, muscular, or neuronal injuries/defects.

Twenty elite soccer players were enrolled. Ten players had no previous HSI (mean ± SD; age: 25 ± 4 years, height: 1.82 ± 0.7 m, and weight: 78 ± 6 kg) and ten players had suffered an HSI during the previous three seasons with sprint-related patterns (mean ± SD; age: 27 ± 4 years, height: 1.84 ± 0.05 m, and weight: 77 ± 6 kg). Injured players had received diagnoses of functional disorder (20%), muscle injury level I (30%), muscle injury level II (40%), and muscle injury level III (10%). They returned to training with the team after the following periods for mild (4–7 days, 20%), moderate (8–28 days, 60%) and severe (>28 days, 20%) injury [19,20]. An MRI and/or ultrasonography was used to diagnose a previous HSI. All players with a history of HSI had fully recovered, were released by the medical staff and participated fully in the training and matches a mean of 18 ± 6 months prior to the evaluation. The data were collected as part of the professional duties in which players are continually monitored throughout the season.

### 2.2. Procedure

#### 2.2.1. BFlh Architecture Assessment from Ultrasound Images

A medical professional with twelve years of knowledge and experience in ultrasonography performed a standardized evaluation of the BFlh in the soccer players. The position of the athletes during ultrasonography, the ultrasound technique employed, the points of BFlh measure, the transducer localization, and the assessment of Fl (mm) and pennation angle (°) were analyzed according to the methods of Núñez et al. [6]. We used a 5 mm probe with an imaging depth of 80 mm and GE-LogiQ S7 (GE Healthcare, Milan, Italy) and ImageJ (ImageJ 1.52p, National Institutes of Health, Bethesda, MD, USA) for data processing and the digitization of muscle thickness (cm), as described in previous research [16]. A very high intra-day repeatability was shown for muscle thickness, pennation angle and Fl (r > 0.90).

#### 2.2.2. Muscle Stiffness Measurement

Players lay prone in a comfortable state on an examination table during the assessment of the passive muscle stiffness of BFlh with a myotonometry device: the MyotonPRO (Myoton AS, Estonia and Myoton Ltd., London, UK). Stiffness is defined as the resistance to a predefined external force that deforms (i.e., stretches or compresses) the muscle’s original shape (i.e., length), and was determined from the mechanical oscillation of the muscle registered by an accelerometer [21]. Measures have been reported to have a high sensitivity to systemic changes [22], validity [21] and relative or absolute reliability [23]. According to standard procedures, landmarks, which included the proximal and distal position of each BFlh, were defined as the regions of interest by ultrasonography.

The mean of a total of 10 measures was used for the analysis (2 sets of 5 consecutive repetitions of the test). The distal and proximal position where stiffness was measured was determined by ultrasound imaging, as previously described.

#### 2.2.3. Eccentric Hamstring

An isokinetic dynamometer and NHE (Nordbord, Vald Performance, Brisbane, Australia) were used to measure the eccentric force of the hamstring. After a warm-up of 10 min cycling in a bicycle ergometer (50 W) and six submaximal trials in the isokinetic dynamometer (Human/NormTM, CSMi, Stoughton, MA, USA), the maximal eccentric knee extensor torque of both knee flexors was measured. For measurements, we followed the procedure described by Aagaard et al. [24]. The rotational axis of the dynamometer was aligned to the subject’s lateral femoral epicondyle. The power leg was attached to the lever arm of the dynamometer above the medial malleolus. The ankle joint could move freely. The measurements were performed from 0° (knee extension) to 90° (perpendicular knee flexion) and at two angular velocities: 30°/s and 120°/s. Two maximal trials were performed with 1 min of rest between them. Subjects were allowed to take a 3 min rest between each velocity. The reliability and validity of the dynamometer and this procedure have been described previously [24]. The participants of this study had not executed NHE systematically before or after the injury.

#### 2.2.4. Nordic Hamstring Exercise (NHE)

A minimum of four days after the isokinetic test, we executed the assessment of NHE strength according to the procedure published by Roe and colleagues [25]. NHEs were executed in field testing according to Roe et al. [25] and the Nordbord software (Nordbord, Vald Performance, Australia). The testing procedures were the same as in previous studies [25,26]. The athletes performed a standardized warm-up including various mobility exercises, active stretching exercises with an emphasis on the posterior chain, and three repetitions of NHE gradually increasing in effort up to the maximum. Following this, players performed 3 repetitions with correct adherence to the proper technique with 30 s of recovery between repetitions. The highest peak force (NHEs_best_) was selected for further comparison, and the peak force for each of the players was averaged for statistical analysis (NHEs_mean_). For analysis, the athletes executed three NHE repetitions with maximal voluntary effort with 30 s of rest between the repetitions. The best (NHEsbest) and mean (NHEsmean) peak forces were used for the analysis.

### 2.3. Statistical Analyses

A one-way analysis of variance (ANOVA) was used to test the effect of a previous HIS on Fl, pennation angle, muscle thickness, stiffness, and eccentric hamstring strength. The normality of the data was evaluated with the Shapiro–Wilk test, which confirmed that the data followed a normal distribution. If the assumption of sphericity as measured by Mauchly’s sphericity test was violated, the Greenhouse–Geisser correction was used. The level of significance was set to *p* < 0.05, and statistically significant differences were marked in data sets with an asterisk (*). We performed a post hoc analysis using Student’s t-tests for pairwise comparisons with a Bonferroni adjustment. Inferential statistical analysis was completed by the estimation of the effect size (ES) using Cohen’s d to evaluate the magnitude of differences [26]. The threshold values for the Cohen’s d were <0.2 (small), <0.6 < (moderate), and >1.2 (large) [27].

Analyses were carried out using JASP statistical software (JASP Team, 2020, v.0.12.0, Amsterdam, The Netherlands) for Windows. Data are presented as mean ± SD.

## 3. Results

Comparisons of muscle architecture and passive muscle stiffness between the injured players and uninjured players are presented in Table 1. Comparisons between the limb with a previous injury and the uninjured limb in injured players revealed no significant differences in muscle architecture and stiffness. When comparing the injured limbs to the limbs of uninjured players, Fl was significantly shorter ((F = 3.676, *p* = 0.039) Injured Limb vs. Right Limbs: *p* = 0.03, ES = 1.0; Injured Limb vs. Left Limbs: *p* = 0.02, ES = 1.06 ) and stiffness was significantly greater ((F = 3.743, *p* = 0.038) Injured Limb vs. Right Limbs: *p* = 0.04, ES = 0.98; Injured Limb vs. Left Limbs: *p* = 0.04, ES = 1.28) at the proximal area in previously injured limbs.

Eccentric knee flexor strength values for players with a previous HSI and for uninjured players are displayed in Table 2. There were no significant differences in NHE and isokinetic eccentric strength between the prior strained limbs and the uninjured limbs among injured players. When comparing the injured limbs to the limbs of uninjured players, there were no significant differences in NHE eccentric strength. Limb dominance had no significant effect on all recorded variables (see Table 3 and Table 4).

## 4. Discussion

This study provides holistic understanding about the architectural characteristics and force profile of the hamstring musculature in elite soccer athletes as related to unilateral HIS histories. The main findings of the present study were: (1) there were no statistical differences in muscle architecture, stiffness, NHE and isokinetic eccentric force between prior strained legs and uninjured legs in the injured players; (2) the FLs were statistically shorter and muscle stiffness was significantly greater at the proximal area of the BFlh in both legs (injured and uninjured) of players with a history of HIS than in the legs of uninjured players; and (3) limb dominance had no significant effect on all recorded variables.

Recent evidence shows that a more flexible musculotendinous system (and less stiffness) has a larger capacity to elongate, allowing external forces to be absorbed and creating a mitigating effect [28]. Previous studies showed that relatively longer muscle fibres can diminish the risk of injury by exposing the sarcomere to less stress with reference to muscle–tendon unit strain [14,16]. In accordance with both aspects, the results of the present study showed that elite soccer players without a history of HIS had significantly longer Fls than players who had suffered a BFlh injury. Due to the non-uniform nature of BFlh architecture [29], a minimum Fl to eliminate the risk of injury cannot be determined. Previous articles suggest that elite soccer players with shorter BFlh Fl (<105 mm) have a much higher risk of sustaining a hamstring injury than players with longer fascicles [5]. The Fls of uninjured elite soccer players in the present investigation was ~9.4 cm, which is in line with similar cohorts (~93 mm) [6], and slightly longer than those detected in soccer players <19 y (~85 mm) [17]. Therefore, there are contradictory results not supporting the idea that soccer players with fascicles <105 mm have a higher risk of suffering a hamstring injury. It is known that soccer training and games induce larger increases in concentric than eccentric force in the BFlh [30], favoring a lengthening of the fascicles of this musculature rather than a shortening [31,32]. However, we also have to take into account which technique was used to measure the Fl, because technique can have a significant influence on the results [16]. The current findings showed that the Fl of limbs with a previous HIS was ~8.3 cm, with no differences from the uninjured limb (8.4 cm). This result disagrees with a previous study, which asserted that a previously injured BFlh had shorter fascicles than the non-injured contralateral leg in multisport samples [18]. Despite variations in cohorts, the lineal extrapolation of fascicles from an individual ultrasonography image used by Timmins et al. [18] (Fl injury limb: 10.4 cm; uninjured limb: 11.9 cm) could result in an overestimation of Fls in comparison with extended field-of-view (EFOV) scans, as used in our study [16]. Likewise, a recent study employing EFOV ultrasound scans demonstrated no differences in Fl between previously injured and non-injured legs in elite soccer players [6]. Therefore, it is not possible to confirm that previously injured BFlh have shorter fascicles and that as a result the BFlh muscle will have a higher risk of suffering a consequent injury. The increased risk to the previously injured leg in injured players will be determined by other risk factors, but not Fl, within this cohort of elite soccer players with previous injuries. Our results also showed longer fascicles in players without previous injuries. Hence, increased Fl may minimize the risk of injury [5,14,16], and further research is needed to track specific training programs aimed at increasing Fl and to determine the possible impact on injury reduction in professional soccer players.

A history of HSI can increase muscle–tendon stiffness in soccer players, with a higher incidence in the proximal than the distal region of the BFlh [6]. Our results showed that proximal BFlh stiffness was higher in previously injured soccer players than in non-injured players, while for distal BFlh stiffness there were no differences between injured and non-injured players. Previous studies showed that the BFlh is selectively innervated during hip flexion, [33,34,35]. In this regard, we speculated that this could be a probable reason for higher BFlh proximal stiffness in players with a history of hamstring injury, but our results revealed no differences in proximal BFlh stiffness between injured and non-injured limbs.

Recent research shows that a previous BFlh injury can reduce hamstring strength [13]. One possible explanation is that injured players may feel apprehension about suffering pain when producing or sustaining high-level eccentric forces [36]. Studies further highlight that athletes with an HSI history, after return to play, perform prone leg curls for shorter durations than non-injured players and have isometric knee flexion force deficits at 15° [4]. Timmings et al. [18] showed that in elite athletes with a unilateral history of BFlh muscle injury, eccentric knee flexor force using the NHE was reduced in the previously injured legs compared to the contralateral non-injured leg. In our study, in contrast, eccentric knee flexor strength was not statistically different between legs. However, Mendez-Villanueba et al. [37] found that the eccentric strength during the NH exercise in elite soccer players was greater in previously injured legs in comparison with the contralateral non-injured leg, and non-injured legs in uninjured players. Our findings have not revealed strength weaknesses in the previously injured leg, either with the NHE or with the isokinetic test. Similar to the results of Mendez-Villanueba et al. [37], our results showed a trend to higher eccentric strength during the NHE in previously injured legs (ES: 1.07–1.32).

Our findings show that injured legs did not have significantly reduced muscle thickness. Muscle thickness reflects BFlh CSA [38] and represents a growth of myofibrils in parallel, facilitating a greater transmission of force developed through the muscle–tendon component [39]. The lack of muscle thickness differences in previously injured players also supports the finding of no loss of strength in injured limbs in comparison with uninjured limbs with regard to eccentric isokinetic strength and NHE. In line with our results, previous research has not documented differences between injured and non-injured legs in soccer players when evaluating isokinetic and NHE eccentric strength [7]. The cohort analyzed in the present investigation had fully recovered from their HSI with no sequels and were training and competing regularly in their domestic leagues, cup, and European competitions. Another explanation could be that the isokinetic test and NHE are predominantly eccentric knee flexor tests, and it is known that BFlh is selectively recruited during hip joint movements [33,34,35], so assessing eccentric hamstring strength in BFlh may not be the best approach. If the main hamstring injury mechanism is based on fast movements with high eccentric forces on the posterior thigh [12], we believe that the assessment of hamstring strength with these characteristics should be integrated.

### Limitations

The present investigation could have some limitations related to the ultrasonography methods used to assess muscle Fls [40,41,42]. Nevertheless, the technique employed for assessment in our study (EFOV scans) has been suggested as optimal due to its lower Fl estimation and better accuracy compared with the trigonometric equation methods used in previous articles [16]. The sample of subjects was not large, but it is very difficult to collect data from this type of elite population. Only injured players completed the isokinetic test, so we could not show the comparison with non-injured players.

## 5. Conclusions

Elite soccer players with previous HSI showed lower Fl and higher proximal stiffness in the BFlh in both the injured and non-injured legs than players without previous hamstring injuries. However, there were no statistical differences in muscle architecture, stiffness, NHE or isokinetic eccentric force between prior strained legs and uninjured legs in the injured players. This absence of differences in morphological and mechanical properties between limbs in the cohort with a history of HIS indicates the presence of a particular characteristic which would be considered in the design of future investigations and prevention programs.

## Figures and Tables

**Table 1:** Comparative muscle architecture and passive stiffness data between limbs of previously injured players and non-injured players. Data are presented as mean ± SD.

	Previously Injured Player				Non-Injured Player			
	Limb with HSI History (*n* = 10)	Uninjured Limb (*n* = 10)	ES	*p*-Value	Right Limb (*n* = 10)	Left Limb (*n* = 10)	ES	*p*-Value
BFlh Fascicle Length (cm)	**8.18 ± 1.08**	**8.38 ± 0.89**	0.17	0.487	9.41 ± 1.28 *	9.46 ± 1.24 *	0.04	0.259
BFlh Pennation Angle (°)	18.18 ± 6.13	16.92 ± 4.70	−0.19	0.265	17.17 ± 5.64	16.49 ± 4.84	−0.11	0.128
BFlh Muscle Thickness (cm)	2.23 ± 0.31	2.19 ± 0.24	−0.11	0.259	2.42 ± 0.26	2.39 ± 0.27	−0.13	0.122
Proximal BFlh Stiffness (N/m)	**308.3 ± 21.9**	**312.8 ± 28.0**	0.18	0.537	269.4 ± 47.5 *	259.6 ± 45.5 *	0.20	0.372
Distal BFlh Stiffness (N/m)	353.7 ± 36.8	359.4 ± 37.0	0.14	0.487	354.3 ± 42.9	332.2 ± 31.6	0.63	0.172

*: Significant differences (*p* < 0.05) with injured limb; bold numbers indicate significant differences between limbs of one player. ES: effect size; *p*: *p*-value; BFlh: biceps femoris long head.

**Table 2:** Comparative knee flexor eccentric strength between the injured and non-injured players. Data are presented as mean ± SD.

	Player with a History of HSI				Non-Injured Player			
	Limb with a History of HSI (*n* = 9)	Uninjured Limb (*n* = 9)	ES	*p*-Value	Rigth Limb (*n* = 10)	Left Limb (*n* = 10)	ES	*p*-Value
NHEbest (N)	363.9 ± 21.4	348.7 ± 12.9	1.07	0.144	363.3 ± 51.9	356.3 ± 48.4	0.13	0.760
NHEmean (N)	353.3 ± 12.8	334.6 ± 12.8	1.32	0.078	355.8 ± 67.8	342.7 ± 52.7	0.21	0.638
Isokinetic Eccentric Peak Torque 30°/s (Nm)	178.3 ± 31.6	192.4 ± 48.8	0.40	0.214				
Isokinetic Eccentric Peak Torque 120°/s (Nm)	168.0 ± 35.1	185.1 ± 39.6	0.43	0.253				

ES: effect size; *p*: *p*-value; NHE: Nordic hamstring exercise.

**Table 3:** Comparative data between injured dominant limb, injured non-dominant limb, uninjured dominant limb and uninjured non-dominant limb in previously injured players. Data are presented as mean ± SD.

	Limb with a History of HSI				Uninjured Limbs			
	Dominant (*n* = 5)	Non-Dominant (*n* = 5)	ES	*p*-Value	Dominant (*n* = 5)	Non-Dominant (*n* = 5)	ES	*p*-Value
BFlh Fascicle Length (cm)	8.3 ± 1.21	8.22 ± 1.1	0.23	0.630	8.32 ± 1.04	8.53 ± 0.77	0.20	0.680
BFlh Pennation Angle (Degrees)	17.67 ± 5.55	18.7 ± 6.1	0.10	0.822	18.69± 7.27	15.13 ± 2.14	0.39	0.873
BFlh Muscle Thickness (cm)	2.22 ± 0.18	2.16 ± 0.33	0.12	0.790	2.16 ± 0.40	2.29 ± 0.16	0.23	0.620
Proximal BFlh Stiffness (N/m)	310.6 ± 13.97	323 ± 39.75	0.16	0.761	305.5 ± 31.58	304.6 ± 14.1	0.05	0.927
Distal BFlh Stiffness (N/m)	366.8 ± 38	363 ± 47.66	0.11	0.832	337.25± 32.19	356.6± 31.84	0.56	0.340
NHEbest (N)	357 ± 26.51	354.75 ± 8.38	0.82	0.198	360 ± 15.64	344 ± 15.08	1.42	0.066
NHEmean (N)	354.4 ± 29.63	336 ± 12.49	0.92	0.162	351.75 ± 12.6	333.5 ± 14.2	1.33	0.076
Isokinetic 30°/s (Nm)	174.5 ± 25.49	215.75 ± 52.38	0.60	0.407	182 ± 40.52	169 ± 36.71	0.27	0.679
Isokinetic 120°/s (Nm)	167.25 ± 37.67	195.25 ± 33.19	1.80	0.200	168.75 ± 38.17	174.75 ± 47.8	0.29	0.659

ES: Effect size; *p*: *p*-value; BFlh: biceps femoris long head; NHE: Nordic hamstring exercise.

**Table 4:** Comparative data between dominant and non-dominant limbs in uninjured players. Data are presented as mean ± SD.

	Dominant Limb (*n* = 10)	Non-Dominant Limb (*n* = 10)	ES	*p*-Value
BFlh Fascicle Length (cm)	9.44 ± 1.26	9.44 ± 1.25	0.01	0.984
BFlh Pennation Angle (Degrees)	17.17 ± 5.58	16.48 ± 4.92	0.51	0.138
BFlh Muscle Thickness (cm)	2.41 ± 0.25	2.4 ± 0.28	0.25	0.439
Proximal BFlh Stiffness (N/m)	269 ± 47.95	260 ± 45.02	0.28	0.678
Distal BFlh Stiffness (N/m)	342 ± 30.66	344.56 ± 46.63	0.05	0.882
NHEbest (N)	354.36 ± 55.29	365.24 ± 44.05	0.43	0.200
NHEmean (N)	350.12 ± 68.93	348.39 ± 52.16	0.04	0.492

ES: Effect size; *p*: *p*-value; BFlh: biceps femoris long head; NHE: Nordic hamstring exercise.

## Data Availability

Data are available on request from corresponding author (ramona.ritzmann@sport.uni-freiburg.de).
